# Pandemic Influenza A H1N1 2009 Infection versus Vaccination: A Cohort Study Comparing Immune Responses in Pregnancy

**DOI:** 10.1371/journal.pone.0033048

**Published:** 2012-03-22

**Authors:** Barbra M. Fisher, Janice Van Bockern, Jan Hart, Anne M. Lynch, Virginia D. Winn, Ronald S. Gibbs, Adriana Weinberg

**Affiliations:** 1 Department of Obstetrics and Gynecology, Section of Maternal-Fetal Medicine, University of Colorado School of Medicine, Aurora, Colorado, United States of America; 2 Departments of Pediatrics, Medicine, and Pathology, University of Colorado School of Medicine, Aurora, Colorado, United States of America; 3 Novartis Diagnostics and Therapeutics, Emeryville, California, United States of America; University of Hong Kong, Hong Kong

## Abstract

**Background:**

With the emergence of H1N1 pandemic (pH1N1) influenza, the CDC recommended that pregnant women be one of five initial target groups to receive the 2009 monovalent H1N1 vaccine, regardless of prior infection with this influenza strain. We sought to compare the immune response of pregnant women to H1N1 infection versus vaccination and to determine the extent of passive immunity conferred to the newborn.

**Methods/Findings:**

During the 2009-2010 influenza season, we enrolled a cohort of women who either had confirmed pH1N1 infection during pregnancy, did not have pH1N1 during pregnancy but were vaccinated against pH1N1, or did not have illness or vaccination. Maternal and umbilical cord venous blood samples were collected at delivery. Hemagglutination inhibition assays (HAI) for pH1N1 were performed. Data were analyzed using linear regression analyses. HAIs were performed for matched maternal/cord blood pairs for 16 women with confirmed pH1N1 infection, 14 women vaccinated against pH1N1, and 10 women without infection or vaccination. We found that pH1N1 vaccination and wild-type infection during pregnancy did not differ with respect to (1) HAI titers at delivery, (2) HAI antibody decay slopes over time, and (3) HAI titers in the cord blood.

**Conclusions:**

Vaccination against pH1N1 confers a similar HAI antibody response as compared to pH1N1 infection during pregnancy, both in quantity and quality. Illness or vaccination during pregnancy confers passive immunity to the newborn.

## Introduction

Among healthy individuals infected with the influenza virus, pregnant women and infants younger than 6 months of age are at increased risk for serious complications when compared to other groups [1]-[3]. These complications include preterm labor, preterm delivery, and pregnancy loss among pregnant women and pneumonia, dehydration, sinus problems and ear infections in infants [4]. Vaccination is the best method to avoid influenza infection and subsequent complications, and even death, among affected pregnant women and their neonates. In 2009, influenza vaccination was recommended for all women pregnant or planning to become pregnant during influenza season [5], [6]. In addition to protecting the pregnant woman, vaccination also protects the newborn from influenza-related complications. This mode of neonatal acquisition of antibodies is extremely important, since influenza vaccines have poor immunogenicity during the first six months of life [7], [8]. Following maternal vaccination, antibodies are actively transferred from the maternal circulation to the fetus via the placenta, providing passive immunity to the neonate [9], [10].

Pandemic influenza A H1N1 (pH1N1) emerged as a threatening pathogen in April 2009. Its effects were realized both nationally and worldwide, and resulted in remarkable morbidity and mortality for both pregnant women and infants [11]-[13]. During the 2009-2010 influenza season, a monovalent vaccine against influenza A pH1N1 virus was developed and recommended as an adjunct to seasonal influenza vaccination among high-risk groups, which included pregnant women [14]. Consistent with seasonal influenza vaccination recommendations, administration of this vaccine was not intended for children younger than 6 months of age. It was expected that the influenza A pH1N1 vaccination, administered to pregnant women, would confer protection to their neonates similarly to seasonal influenza vaccination [10], [15].

Reports of the immune response to influenza during pregnancy have focused on the antibody response to vaccination. We found no reports of the immune response to wild-type influenza infection during pregnancy in the literature. Here, we characterize the antibody response during pregnancy to influenza A pH1N1 vaccination as well as wild-type infection and demonstrate that passive immunity to the neonate results from provocation of maternal antibody production from either vaccination or infection.

## Materials and Methods

### Patient recruitment

This prospective cohort study was approved by the IRB at the University of Colorado School of Medicine (study 09-0970). All patients gave written consent at time of enrollment in this study and the clinical investigation was conducted according to the principles expressed in the Declaration of Helsinski.

Pregnant women were recruited for this study upon admission to labor and delivery from November 2, 2010 through June 17, 2011. During the 2009-2010 influenza season, the University of Colorado Hospital (UCH) instituted a triage system (influenza triage system) whereby all high-risk individuals with influenza-like illness (ILI), including pregnant women, would be evaluated in person and tested for influenza infection. Based on local and worldwide reports, all circulating influenza A during this influenza season was presumed to be the pandemic H1N1 influenza A strain. Respiratory specimens were obtained from patients and rapid antigen influenza A testing was performed. Based on the low sensitivity of the rapid test, 19% in one study [16], all specimens with negative results had reflex PCR testing performed. All patients with a positive result from either the rapid antigen test or PCR test were presumed to have been infected with the influenza A pH1N1 virus.

The influenza triage system at UCH and subsequent electronic record-keeping of all triaged patients and their accompanying test results allowed us to identify women infected with pH1N1 influenza during pregnancy. For this study, three different groups of pregnant women were identified and recruited (*Infected, Vaccinated, and Control*). The *Infected* group was comprised of women infected with laboratory-confirmed pH1N1 influenza during the current pregnancy, with or without vaccination. The *Vaccinated* group was comprised of women vaccinated against the pH1N1 influenza virus during the current pregnancy based upon review of the medical records and without ILI during pregnancy. The *Control* group was comprised of women reporting neither ILI nor pH1N1 influenza vaccination during pregnancy or preconception during the 2009-2010 influenza season. Individuals in the *Vaccinated* and *Control* groups were matched to individuals in the *Infected* group based on gestational age at delivery, parity, and planned mode of delivery. Women with antepartum pH1N1 infection, vaccination, or neither were identified at time of admission to labor and delivery and consented for study enrollment at that time.

### Specimen collection, processing, and assays performed

At the time of study enrollment, maternal sera were collected, aliquoted, and stored at −80°C. At delivery, umbilical cord venous blood was collected similarly, aliquoted, and stored at −80°C. In batches, hemagglutination inhibition (HAI) assays were performed for paired maternal/umbilical cord venous sera samples, as described previously [17]. Serum dilution started at 1∶10. Sera with HAI titers < 1∶10 were arbitrarily ascribed a value of 1∶5 for calculations of geometric mean titers. The technician performing the HAI assays was blinded to the specimen’s group assignment and whether the specimen originated from maternal or umbilical cord blood.

### Ascertainment of pregnancy characteristics

We used records from the Perinatal Database of the Department of Obstetrics and Gynecology at the University of Colorado School of Medicine to obtain information regarding maternal racial/ethnic group (non-Hispanic white, Hispanic, black, Asian, and other) and pregnancy characteristics (parity and gestational age (based on last menstrual period and ultrasound)). Information on these and other perinatal variables is collected by research assistants on every woman delivering at UCH. This information is entered into a database housed in the Division of Biostatistics and Bioinformatics at National Jewish Health, Denver, CO. During the 2009-2010 influenza season, an H1N1 Questionnaire, an adjunct to the Perinatal Database, was developed and administered to postpartum women delivering at UCH [18]. Clinical information related to ILI dates and symptoms, pH1N1 influenza testing, and pH1N1 influenza vaccination were obtained by administering the H1N1 Questionnaire to all patients enrolled in the current study.

### Statistical analysis

**Figure 1:**
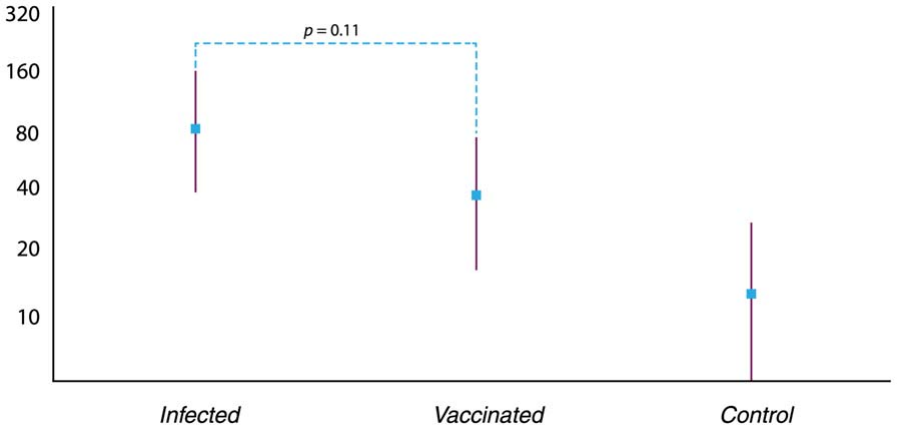
Geometric mean titers for the cohort.

**Figure 2:**
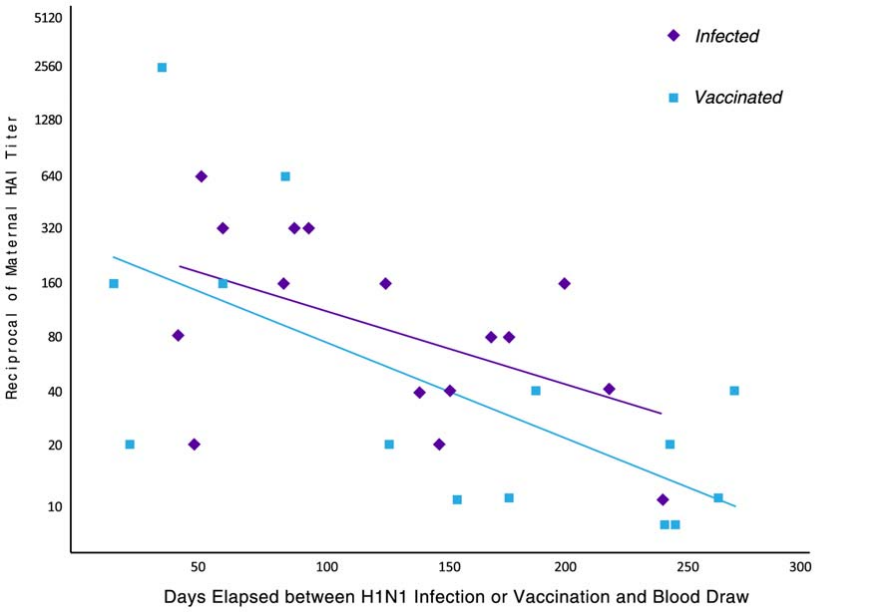
Persistence of maternal antibodies to pandemic H1N1 influenza following wild-type infection or vaccination.

The data were analyzed in SAS 9.2 (SAS Institute, Cary, NC). Linear regression models were used to investigate the antibody response during pregnancy to influenza A pH1N1 vaccination and wild-type infection and investigate passive immunity to the neonate. Comparisons among groups were considered significant if *p* < 0.05. When expressing reciprocal antibody titers, the logarithm of the antibody titer was used to best fit linear equations; for Figures 1 and 2, the reciprocal antibody titer is shown on the relevant axes.

## Results

### Characteristics of the study population

We enrolled 40 women in this study: 16 women with confirmed pH1N1 infection during pregnancy (*Infected)*, 14 women with documented pH1N1 vaccination during the pregnancy (*Vaccinated*), and 10 control women (*Control*) who were neither infected nor vaccinated. Of the 16 women in the *Infected* group, four were also vaccinated against pH1N1 influenza. One woman was vaccinated three days prior to presenting with influenza-like illness, and the other three patients were vaccinated between five and seven weeks following confirmed pH1N1 influenza infection. All analyses were performed with both inclusion and exclusion of these four patients in the *Infected* group; since there were no statistical differences between results with either inclusion or exclusion of these four patients, we opted to keep them included in the *Infected* group. We compared the characteristics of the *Infected* women, the *Vaccinated* women, and the *Control* women (Table 1). At study enrollment, matching based on gestational age at delivery, parity, and planned mode of delivery was performed. As shown in Table 1, the groups were indeed similar with respect to these characteristics. In addition, the groups did not differ with respect to maternal age, gestational age at time of pH1N1 infection or vaccination, or race/ethnicity. The only difference among groups was BMI, with the mean BMI in the control group being higher.

**Table 1:** Characteristics of the cohort (n = 40).

	*Infected* (n = 16)	*Vaccinated* (n = 14)	*Control* (n = 10)	*p* value
Maternal Age (years), mean±SD	26.1±3.7	27.4±5.6	25.6±4.6	0.62
Race/Ethnicity, n (%)				
Hispanic	3 (18.7)	5 (35.7)	4 (40.0)	0.17
White	9 (56.3)	9 (64.3)	3 (30.0)	
Other/mixed	4 (25)	0 (0.0)	3 (30.0)	
BMI, mean±SD	26.0±4.6	24.9±5.6	31.9±10.1	0.04
Gestational age at delivery (wks), mean±SD	39.1±2.1	39.4±1.0	39.8±1.4	0.57
Gestational age at H1N1 infection or vaccination (wks), mean±SD	21.7±10.5	17.7±13.1	n/a	0.37

### HAI antibody titers in women with pH1N1 wild-type infection or vaccination during pregnancy

In Figure 1, we show the geometric mean titers (GMT) for the *Infected*, *Vaccinated*, and *Control* groups of women. The GMT following pH1N1 infection or vaccination is similar, with the *Infected* and *Vaccinated* groups differing from the GMT for the *Control* healthy, non-vaccinated, patients. Four subjects in the *Infected* group received the vaccine during pregnancy (three of which received vaccination after confirmed infection), in spite of documented wild-type infection as per the recommendations for universal influenza vaccination during pregnancy [6]. The GMT at delivery for these four subjects was 1∶160, which did not differ statistically from the GMT at delivery for the non-vaccinated *Infected* patients (1∶71; *p* = 0.32).

HAI antibody titers ≥ 1∶40 are considered to be relevant clinically, and are considered to result in a 50% decrease in symptomatic infection [19]. Whether a woman had confirmed pH1N1 infection or vaccination, the mean titer was at, or greater than, this threshold value, suggesting sufficient immunity toward the pH1N1 influenza virus. Control patients, individuals who had neither pH1N1 infection nor vaccination, had HAI titers well below this threshold value, suggesting no immunity toward pH1N1 influenza.

Influenza-specific antibodies after vaccination are typically short-lived. To determine if antibody decay differed between wild-type infection or vaccination during pregnancy, we evaluated the relationship between the maternal HAI titers and days elapsed between pH1N1 infection or vaccination and blood draw at time of admission to labor and delivery (Figure 2). There is a linear correlation between these titers and days elapsed between pH1N1 infection or vaccination for both groups. In this figure, we demonstrate that there was a significant linear decline over time in HAI titers after pH1N1 infection or vaccination (*p* = 0.04 for *Infected* and *p* = 0.009 for *Vaccinated*). Furthermore, the rate of decay of pH1N1 antibodies, measured by the slope of the HAI titers over time, was similar for antibodies produced in response to wild-type infection or vaccination (−0.010 and −0.013, respectively; *p* = 0.60). A sensitivity analysis performed by censoring the data of the four women who were both vaccinated and had wild-type infection showed similar results.

### Transplacental transfer of pH1N1 antibodies after wild-type infection or vaccination during pregnancy

**Figure 3:**
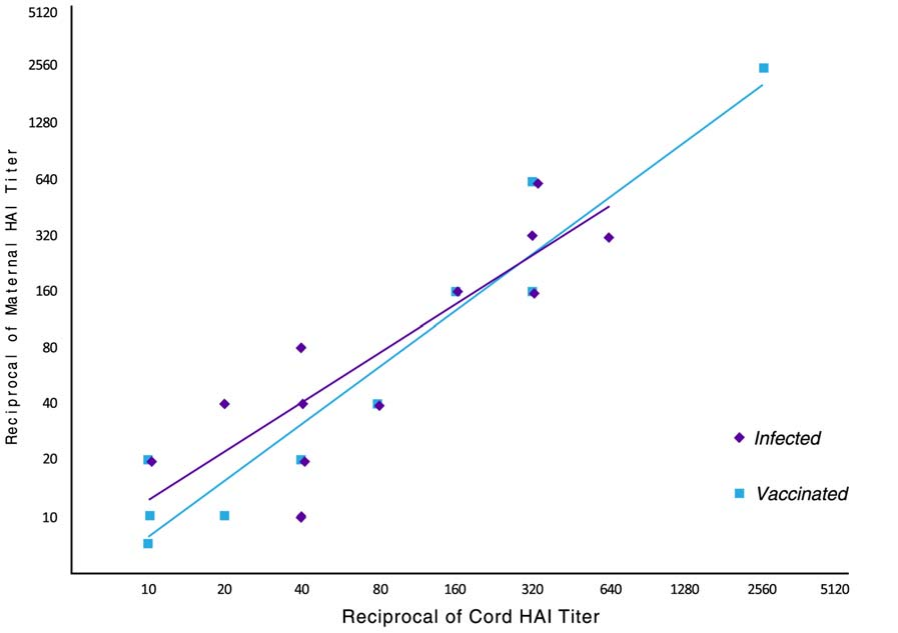
Transplacental transfer of antibodies to pandemic H1N1 influenza following wild-type infection or vaccination.

In Figure 3, we show the relationship between maternal and cord blood HAI titers. There was a linear relationship between maternal and cord HAI titers after wild-type infection or vaccination (R^2^ = 0.76 for *Infection* and R^2^ = 0.92 for *Vaccinated*), demonstrating efficient transplacental transfer of pH1N1 influenza antibodies. Further, this efficient transplacental antibody transfer was similar for both groups (*p* = 0.85).

## Discussion

In this cohort, we demonstrate that pregnant women exposed to either wild-type pH1N1 infection or pH1N1 vaccination produce antibodies sufficient to provide short-term protection against homotypic influenza infection. An HAI antibody titer of 1∶40 after vaccination is the current standard for licensure of influenza vaccines and a widely accepted surrogate for protection against influenza infection [19]. In this study, women who were infected with or vaccinated against pH1N1 had GMTs during pregnancy above this threshold value, suggesting protection against this specific influenza strain.

It has been shown that antibodies against one influenza virus type or subtype confer limited or no protection against other types or subtypes of influenza virus [20]. In a typical influenza season, when there may be three or four influenza strains circulating in the community, documented influenza infection does not preclude the need for vaccination, which may confer protection against additional strains. However, in a season such as 2009-2010 when a monovalent preparation is used to protect against a single influenza strain, confirmed infection should negate the need for strain-specific vaccination, as shown by the similar antibody response to either infection or vaccination. Four of our patients with confirmed pH1N1 infection were also vaccinated, according to the recommendations of the CDC. These four patients had a non-significant increased (2-fold) antibody titer when compared to either the *Infected* or *Vaccinated* patients. After analyzing our data with both the inclusion and exclusion of these four patients, we found that their inclusion in the *Infected* group did not change the results reported herein.

Although pH1N1 infection and vaccination resulted in similar HAI antibody titers, these results do not completely establish that the level of protection afforded to an individual, following either infection or vaccination, are the same. For example, elderly individuals are less likely to develop influenza illness if they had infection rather than vaccination in the preceding season [21]. Similarly, the incidence of infection with pH1N1 in 2009-2010 was higher in elderly recipients of the 2008-2009 seasonal influenza vaccination compared with unvaccinated individuals of the same age [22]. The inactivated vaccine generates much lower cell-mediated immune responses compared with wild-type infection. The influenza-specific cell-mediated immunity, and particularly granzyme B-producing cytotoxic T-lymphocytes, appear to be critical for protection against disease [23]. It also confers heterosubtypic cross-protection, since there is higher similarity among T-cell compared with B-cell epitopes among different influenza serotypes [24], [25]. Live attenuated vaccines tend to generate higher levels of cell-mediated immune responses that do inactivated preparations. However, they are contraindicated in pregnancy. The use of adjuvants may also increase cell-mediated immune responses to inactivated vaccines and deserve to be studied further.

We show that antibody titers decline over time, and this decline is similar whether antibodies are produced in response to wild-type pH1N1 infection or pH1N1 vaccination. This decline could have clinical significance for an influenza season with atypical timing, such as the 2009-2010 pH1N1 influenza outbreak. In contrast to typical influenza seasons where the population is immunized in the fall months with peak influenza season activity seen during the subsequent winter months, the H1N1 pandemic began during springtime, peaked during the summer, persisted through the following winter, and reappeared in the subsequent influenza season. Here, at approximately 150 days for the vaccinated women and 225 days for the infected women, the line of best fit for the antibody titer in response to vaccine or infection crossed below the 1∶40 antibody titer threshold. For select women in this cohort who received vaccination early in pregnancy, greater than 150 days elapsed between vaccination and delivery. This observation lends the question: should repeat vaccination for an atypically-timed influenza strain be encouraged for pregnant women vaccinated early in pregnancy? Further, these data support national recommendations for yearly influenza vaccination.

Vaccine administration to pregnant women has been used to protect infants against infection in the first few months of life. The best example of success in this strategy is the dramatic decline in the incidence of neonatal tetanus in response to maternal immunization [26], [27]. A similar strategy could be used to prevent influenza in young infants who are at high risk of developing severe disease if infected with influenza, but in whom current vaccines have poor immunogenicity. Indeed, Zaman *et al*. demonstrated that inactivated influenza vaccine administered to pregnant women resulted in a 63% reduction in laboratory-proven infection in infants up to 6 months of age and reductions of 29% and 36% in rates of respiratory illness with fevers in infants and mothers [9]. Subsequent independent studies confirmed these results [10]. The decreased incidence of influenza in infants after maternal immunization could result from passive immunity acquired through transplacental transfer of maternal antibodies and/or from decreased exposure to maternal influenza infection. Here, we examined transplacental antibody transfer following pH1N1 vaccination. Administration of the 2009 monovalent pH1N1 vaccination to pregnant women resulted in detectable antibodies in umbilical cord venous blood with GMTs ≥ 1∶40. This finding is consistent with previous studies with seasonal influenza vaccination [28]. Notably, following pH1N1 wild-type infection, a similar linear relationship between maternal and umbilical cord antibody titers was observed.

The main limitation of this study was the small number of participants. Despite gathering a research team rapidly and obtaining IRB approval for this study in a timely fashion, we were limited by the relatively small number of women at our institution with confirmed pH1N1 infection or vaccination during pregnancy and to the difficulty of enrolling patients who were identified as potential study candidates during labor. Another limitation of this study is that baseline antibody titers, prior to vaccination administration, were not available in the vaccination group. It is possible that some of the women in this group had previous asymptomatic infection, and that prior infection might have bolstered the immune response, affecting the comparison between the vaccinated and infected groups of women. Each woman in this study, prior to enrollment, was interviewed to insure no pH1N1 infection during pregnancy. Among the 813 women that we screened for study enrollment, 122 reported symptoms consistent with influenza-like illness and were tested for H1N1 infection. Of these women, only 38 (38/813 = 5%) had confirmed pH1N1 infection (Fisher *et al*., data presented at the 2009 IDSOG annual meeting). Assuming that 25-30% of the pH1N1 infections were asymptomatic [29], [30], a conservative estimate would be that up to 2% of the participants in the vaccination group may have had asymptomatic infection in addition to vaccination. Hence it is unlikely that asymptomatic infection biased our results.

In summary, this study demonstrates that vaccination against pH1N1 confers a similar antibody response as compared to pH1N1 infection during pregnancy, in quantity, quality, and persistence. In addition, both illness and vaccination during pregnancy confers passive immunity to the newborn. These findings support repeat vaccination of pregnant women during each influenza season, regardless of infection or vaccination in the previous season, but do not support administration of monovalent influenza vaccines to pregnant women who already experienced infection with the same vaccine strain in the same season. In future influenza pandemics where a monovalent vaccination is available, guidelines for vaccination of individuals with prior confirmed infection should be re-evaluated. In addition, for atypically-timed influenza outbreaks (such as that seen during the 2009-2010 H1N1 influenza pandemic), where a woman might be vaccinated during the first trimester and influenza is still circulating months later at time of delivery, a booster dose might be considered for the benefit of the neonate. This recommendation warrants further investigation.
